# The Defeat of the Osteopathy Bill

**Published:** 1901-01

**Authors:** 


### THE DEFEAT OF THE OSTEOPATHY BILL.

THE bill to regulate the practice of osteopathy in the State of Georgia was defeated in the senate by a vote of 18 to 19, a constitutional majority being required for its passage. The defeat of the bill means that the Governor's veto last year has been sustained by the judgment of the General Assembly, and that this State is not to be included among those in which the official imprimatur has been placed upon a raw and crude quackery. The history of civilization has shown the rise and fall of numberless fads in the treatment of disease, and the same phase of human nature which rendered man gullible in the past persists and will doubtless extend into the future. The argument that overcomes them all is the fact that disease is not a fixed condition nor does it arise from one common cause, and hence he who essays to arrest its course or prevent its occurrence must have many and varied means of so doing—must play on stops of various quills. There is no all-healing plant, there is no opopanax for the healing of the nations. This is an inexorable logic and one that is sure to cause the overthrow of any system which pretends to meet and fight disease with one weapon. We may never find the solution of the problem that concerns the arrest of that decay which surely fastens upon every created thing. All that lives must die, passing through nature to eternity, and seekers after the truth may find it in the general eclecticism that utilizes all obstructive and corrective agencies, and the clear path of progress stretches away in the elaboration and elucidation of these agencies. Every creed and cult and sect that is controlled by a single idea may be properly relegated to the limbo of the utopian and the useless.

It is hoped that we have seen the last in this State of this particular claimant of popular credulity.

---

OFFICERS OF THE ATLANTA MEDICAL SOCIETY.—The officers elected for 1901, were the following: President, T. Virgil Hubbard; Vice-President, Bernard Wolff; Secretary, Claude A. Smith; Treasurer, E. Van Goidtsnoven. Dr. L. B. Clarke was elected one of the executive committee to fill the vacancy created by the expiration of Dr. R. R. Kime's term.

---

THREE ex-presidents of the American Medical Association died within a week, in September. These were Drs. Hunter McGuire, Lewis A. Sayre and Alfred Stillé. As the *Journal of the Association* notices, all were advanced in years: McGuire was sixty-five, Sayre eighty, and Stillé eighty-seven. Of former presidents of the Association eleven still survive: N. S. Davis, 1864–5, age eighty-three; Elisha H. Gregory, 1887, age seventy-six; Edward Mott Moore, 1890, age eighty-six; Henry O. Marcy, 1892, age sixty-three; James F. Hibberd, 1894, age eighty-four; Donald Maclean, 1895, age sixty-one; Beverly R. Cole, 1896, age seventy-one; Nicholas Senn, 1897, age fifty-six; George M. Sternberg, 1898, age sixty-two; Joseph M. Matthews, 1899, age fifty-three; and W. W. Keen, 1900, age sixty-three.—*Med. Standard.*

